# The effect of tumor composition on the success of adaptive therapy: The case of metastatic Castrate-Resistant Prostate Cancer

**DOI:** 10.1371/journal.pone.0308173

**Published:** 2024-09-26

**Authors:** Monica Salvioli, Len Vandelaer, Esther Baena, Katharina Schneider, Rachel Cavill, Kateřina Staňková

**Affiliations:** 1 Evolutionary Game Theory Lab, Faculty of Technology, Policy and Management, Delft University of Technology, Delft, The Netherlands; 2 Department of Advanced Computing Sciences, Maastricht University, Maastricht, The Netherlands; 3 Cancer Research UK Manchester Institute, The University of Manchester, Manchester, United Kingdom; University of Health and Allied Sciences, GHANA

## Abstract

Prostate-specific antigen (PSA) is the most commonly used serum marker for prostate cancer. It plays a role in cancer detection, treatment monitoring, and more recently, in guiding adaptive therapy protocols, where treatment is alternated based on PSA levels. However, the relationship between PSA levels and tumor volume remains poorly understood. Empirical evidence suggests that different cancer cell types produce varying amounts of PSA. Despite this, current mathematical cancer models often assume either that all cell types contribute equally to PSA levels or that only certain subpopulations produce PSA at fixed rates. In this study, we compare Zhang et al.’s classical adaptive therapy protocol with the standard of care, which involves continuous maximum tolerable dose treatment, under different assumptions regarding PSA production. Specifically, we explore the possibility that testosterone-dependent, testosterone-producing, and testosterone-independent cells contribute to PSA production to varying degrees. We use the time to competitive release as a proxy for the time to disease progression. Our findings indicate that adaptive therapy consistently results in a longer time to competitive release compared to the standard of care, regardless of the assumptions about PSA production. However, when testosterone-independent cells are the sole PSA producers, Zhang et al.’s adaptive therapy protocol becomes inapplicable, as PSA levels never fall to half of their initial value, preventing therapy discontinuation. Additionally, we observe that the number and duration of treatment cycles in adaptive therapy are highly sensitive to assumptions about how much each cell type contributes to PSA production. Overall, our results emphasize the need for a deeper understanding of patient-specific PSA dynamics, which could enhance the effectiveness of adaptive therapy in prostate cancer treatment.

## Introduction

Prostate-specific antigen (PSA) is an enzyme produced by both normal and cancerous prostate epithelial cells [1]. The blood PSA levels are influenced by many factors, including the age of the patient, the ethnic group, the size of prostate, the presence of prostate cancer and its stage and tumor volume [2–4]. For this reason, the precise relationship between the PSA level and the tumor volume remains poorly understood [5–7]. Nevertheless, PSA is currently the most widely used serum marker to diagnose, stage and monitor prostate cancer, and to assess responses to treatment [1, 8–10]. Getting information on tumor response to treatment and progression became even more crucial with the advent of new treatment strategies such as adaptive therapy (AT), which modulates the treatment depending on the response of the specific patient [11, 12].

In a pilot clinical trial (NCT02415621) applying AT to patients with metastatic Castrate-Resistant Prostate Cancer (mCRPC), decisions were guided entirely by the level of PSA. Patients were treated with the CYP17A inhibitor abiraterone acetate, to lower testosterone auto-production. Patients who did not respond to a low initial dose of abiraterone were excluded from the trial, while responders received maximum tolerable dose of abiraterone until their PSA levels dropped to 50% or less of the initial value. At this point, abiraterone was discontinued until PSA returned to the baseline. Each patient followed this cycle of treatment being adaptively turned on and off based on their PSA levels until radiographic progression occurred [12]. The trial demonstrated that adaptive dosing more than doubled the time to progression compared to the standard of care, which involved continuous abiraterone at the maximum tolerable dose (MTD). The median time to progression was approximately 30 months with adaptive dosing, compared to about 14 months with the standard of care [13, 14].

The AT protocol was derived from a game-theoretic model of metastatic Castrate-Resistant Prostate Cancer, utilizing three competing cancer cell types: *T*^+^ cells requiring exogenous testosterone to survive, *T*^*P*^ cells producing testosterone due to the upregulation of the enzyme CYP17A, and testosterone-independent *T*^−^ cells [12, 15]. Zhang et al. (2017) assumed that each of these cell types produces one unit of PSA and that 50% of the PSA decays out of the blood serum per unit time:

$$\begin{array}{c} \frac{d}{dt}\text{PSA}\left(t\right)=\sum_{i\in T}x_{i}\left(t\right)-0.5\cdot \text{PSA}\left(t\right), \end{array}$$ (1)

with $T=\{T^{+},T^{P},T^{-}\}$ and *x*_*i*_ being the number of cells of the corresponding type [12].

The PSA dynamics have been explored in detail in many mathematical models. For instance, West et al. (2019) used the same assumptions of Zhang et al. (2017) and extended the formula to model four different cancer cell types [16]. Hansen et al. (2020) kept the 50% decay rate but assumed that each cell type produces two units of PSA per time unit [17]. As it is unclear how precisely the PSA level decays, Cunningham et al. (2018 and 2020) did not assume any decay rate but assumed that PSA simply measures $\sum_{i\in T}x_{i}$ [18, 19].

Hirata et al. (2010) considered three slightly different cell types: androgen-dependent cells, androgen-independent cells resulting from reversible changes, and androgen-independent cells arising from irreversible changes of genetic mutations. Still they assumed that each type produces one unit of PSA without any decay, similarly to other works [20–26].

Brady-Nicholls et al. (2020 and 2021) proposed a model of prostate cancer stem cells and non-stem prostate cancer cell dynamics to simulate the observed PSA response patterns [27, 28]. In this approach, only differentiated non-stem cancer cells are simulated to produce PSA with a fixed rate, while stem cells do not. The model was calibrated and validated using patient-specific data from two different clinical trials, which tracked PSA decay during treatment and PSA increase during treatment holidays [12, 29].

*In vitro* experiments by Gustavsson et al. (2005), who cultured an androgen-dependent human prostate cancer cell line until an androgen-independent sub-line emerged and measured the corresponding PSA secretion, suggest that cell types may contribute differently to PSA production [30]. This would mean that the PSA dynamics, as introduced in [12, 18], might not reflect the actual tumor burden and that a more precise estimation of the PSA could be derived by accounting for the heterogeneity of the tumor cell population.

Consistent with this finding, we build on the Zhang et al.’s model, which guided their trial [12, 14]. Within each cell type, we assume that all cells produce identical amount of PSA, but we assume that the three cell types can produce different amounts of PSA. We use the model to explore various scenarios, including cases where *T*^+^ cells, *T*^*P*^ cells, or *T*^−^ cells are the primary producers of PSA, as well as intermediate cases. Specifically, we aim to investigate and model how these different PSA production assumptions impact the effectiveness of AT.

In the next section, we introduce the model and its parameters. Building on previous work [12, 15, 18, 19], we consider three categories of patients, based on their treatment response: best responders, responders and non-responders. We then compare the success of the treatment for each patient category under AT versus continuous MTD, considering variations in PSA production by different cell types. We conclude by summarizing the findings and discussing the study’s limitations and potential directions for future research.

## Materials and methods

We use the Lotka-Volterra competition model by [12, 18, 19] to describe the interactions between the testosterone-dependent *T*^+^, the testosterone-producer *T*^*P*^ and the testosterone-independent *T*^−^ cell types under abiraterone therapy. The instantaneous rate of change in the population size of each cell type $i\in T=\{T^{+},T^{P},T^{-}\}$ is:

$$\begin{array}{c} \frac{dx_{i}}{dt}=r_{i}x_{i}(1-\frac{\sum_{j\in T}a_{ij}x_{j}}{K_{i}}), \end{array}$$ (2)

where *r*_*i*_ represents the growth rates, *K*_*i*_ the carrying capacities and *a*_*ij*_ the coefficients of the competition matrix

$$A=(a_{i,j})=\begin{array}{ll} \;\;\;\;T^{+}\;\;\;\;\;\;\;T^{P}\;\;\;\;\;\;T^{-} & \\ \left(\begin{array}{lll} a_{1,1} & a_{1,2} & a_{1,3}\\ a_{2,1} & a_{2,2} & a_{2,3}\\ a_{3,1} & a_{3,2} & a_{3,3} \end{array}\right) & \begin{array}{c} T^{+}\\ T^{P}\\ T^{-} \end{array} \end{array}.$$ (3)

As per Zhang et al. (2017), Cunningham et al. (2018), and Cunningham et al. (2020), we set the growth rates to $r_{T^{+}}=0.0027726$, $r_{T^{P}}=0.0034657$ and $r_{T^{-}}=0.0066542$. These growth rates are derived from the measured doubling times of representative cell lines: LNCaP (ATCC@CRL-1740) for *T*^+^, H295R (ATCC@CRL-2128) for *T*^*P*^ and PC-3 (ATCC^®^CRL-1435) for *T*^−^ [12, 18, 19, 31].

Following [12, 18, 19], we assume that abiraterone reduces the ability of *T*^+^ and *T*^*P*^ cells to acquire testosterone and we model this effect as a reduction in the carrying capacity of these cell types. In particular, abiraterone diminishes the ability of *T*^*P*^ cells to exploit the CYP17A pathway to convert cholesterol into testosterone and other androgens and therefore inhibits the production of testosterone. For this reason, in the absence of treatment the carrying capacity of the *T*^*P*^ cells is set to $K_{T^{P}}=10000$, while under treatment it is reduced to $K_{T^{P}}=100$. As the *T*^+^ cells rely on the endogenous testosterone produced by the *T*^*P*^ cells [12, 18, 19], we assume that their carrying capacity is a linear function of the density of the $T^{P}:K_{T^{+}}=\mu x_{T^{P}}$, where *μ* = 1.5 in the absence of therapy and *μ* = 0.5 under therapy as in [12]. As the *T*^−^ cells are not affected by abiraterone, their carrying capacity is always $K_{T^{-}}=10000$.

Each competition coefficient *a*_*i*,*j*_ describes the effect of cells of type *j* on the growth rate of cells of type *i*. The intra-cell type coefficients are set to *α*_*i*,*i*_ = 1. Zhang et al. (2017), You et al. (2017), Cunningham et al. (2018), and Cunningham et al. (2020) assumed that the inter-cell type coefficients have values from the set {0.4, 0.5, 0.6, 0.7, 0.8, 0.9}. They distinguished 22 cases, which they group into three categories, depending on the frequency of *T*^−^ cells at the equilibrium [12, 15, 18, 19]:

- **Best responders**: twelve cases with a competition matrix promoting the absence of *T*^−^ and high frequencies of both *T*^+^ and *T*^*P*^. Like Cunningham et al. (2018) we use the following representative competition matrix for this category to explore model predictions [18]:
  $$\begin{array}{c} A=(\begin{array}{ccc} 1 & 0.7 & 0.8\\ 0.4 & 1 & 0.5\\ 0.6 & 0.9 & 1 \end{array}) \end{array}$$ (4)
- **Responders**: four cases with competition matrices resulting in low frequencies of *T*^−^ at initiation of therapy. Following [18] for this category we use this representative competition matrix:
  $$\begin{array}{c} A=(\begin{array}{ccc} 1 & 0.7 & 0.8\\ 0.4 & 1 & 0.6\\ 0.5 & 0.9 & 1 \end{array}) \end{array}$$ (5)
- **Non-responders**: six cases with a competition matrix resulting in high equilibrium frequencies of *T*^−^ (≥20%). As in [18], for this category we use the following representative competition matrix:
  $$\begin{array}{c} A=(\begin{array}{ccc} 1 & 0.7 & 0.9\\ 0.4 & 1 & 0.6\\ 0.5 & 0.8 & 1 \end{array}). \end{array}$$ (6)

For details about the specific cases, we refer the reader to [15]. The initial cell counts for each category are taken from [18] and reported in Table 1.

**Table 1:** Population densities (cell counts) at time 0 for each subpopulation (*T*^+^, *T*^*P*^ and *T*^−^) and each patient category (best responders, responders, non-responders), The values are taken from [18] and are used as initial conditions for Eq 2.

	$x_{T^{+}}(0)$	$x_{T^{P}}(0)$	$x_{T^{-}}(0)$
**Best responder**	606.06	757.58	1.94⋅10^−10^
**Responder**	560.36	747.59	47.10
**Non-responder**	319.63	707.76	273.97

As opposed to [12, 18, 19], where the PSA level at a certain time *t* is assumed to correspond to the total number of cancer cells at that time up to some decay, here we assume that the three cell types can produce different amounts of PSA, so that the PSA level at a certain time *t* corresponds to:

$$\begin{array}{c} \text{PSA}\left(t\right)=\alpha x_{T^{+}}\left(t\right)+\beta x_{T^{P}}\left(t\right)+\left(1-\alpha -\beta \right)x_{T^{-}}\left(t\right), \end{array}$$ (7)

where *α* and *β* determine the amount of PSA produced by the *T*^+^ and *T*^*P*^, respectively, with 0 ≤ *α* ≤ 1 and 0 ≤ *β* ≤ 1 − *α*. For each representative case, we compare the outcome under continuous MTD to the outcome under AT, where the treatment is administered until the PSA drops to half of its initial value, then discontinued and readministered only when the PSA recovers to its initial level. Following common interpretation [19, 32], we refer to adaptive therapy only if the treatment is discontinued at least once.

In our case studies, we measure the success of the treatment through the time to competitive release (TCR), defined as the time at which *T*^−^ cells become the majority of the tumor composition. Following [12, 18], we define this time as:

$$\begin{array}{c} \text{TCR}=\text{min}\{t\in \left[0,T\right]:x_{T^{-}}\left(t\right)\geq x_{T^{+}}\left(t\right)+x_{T^{P}}\left(t\right)\}. \end{array}$$ (8)

In our model, the treatment is applied even after reaching the TCR. While the TCR under adaptive therapy is influenced by *α* and *β*, the TCR under MTD is not and depends only on the category which we consider (best responders, responders, non responders).

## Results

In the following sections, we compare the effectiveness of the standard of care applying MTD with AT under different assumptions on the PSA production. We present results for the three patient categories: best responder, responder, and non-responder, focusing on four different assumptions on PSA production: 1) all cell types contribute equally to PSA production, i.e., $\alpha =\beta =\frac{1}{3}$, 2) only *T*^+^ cells produce PSA, i.e., *α* = 1, *β* = 0, 3) only *T*^*P*^ cells produce PSA, i.e., *α* = 0, *β* = 1, and 4) only *T*^−^ cells produce PSA, i.e., *α* = 0, *β* = 0. For completeness, we also explore all the intermediate values of *α* and *β*. All calculations and model simulations were performed in Wolfram Mathematica version 13.0.

### Best responders

**Fig 1:**
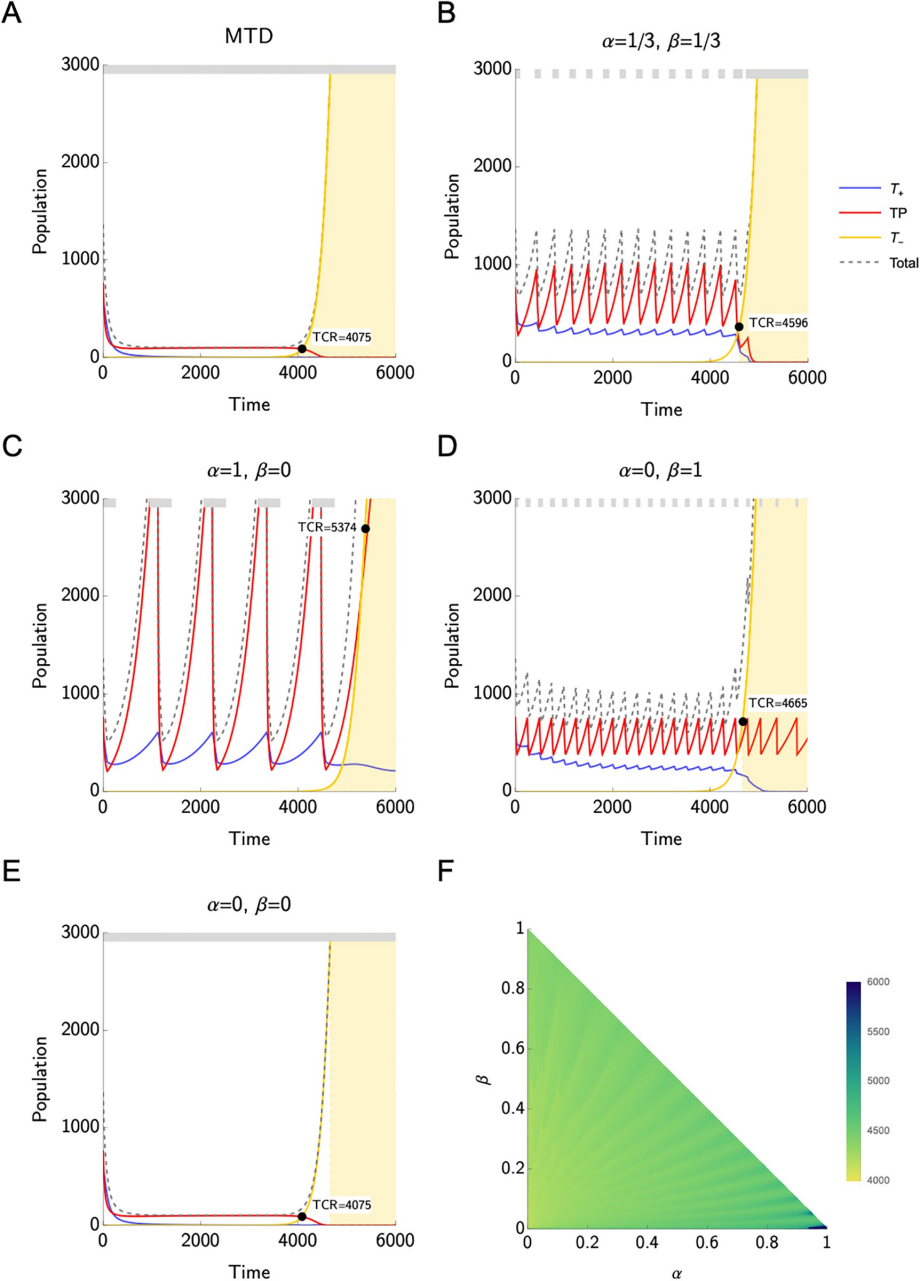
Best responders. **A-E**) Time to competitive release (TCR) under maximum tolerable dose (A and E) and adaptive therapy for different values of *α* and *β* (B-D). The grey bar on the top of each plot indicates when treatment is on. For all the values of *α* and *β* considered here, adaptive therapy is better than the standard of care based on maximum tolerable dose. If *α* = *β* = 0, i.e. *T*^−^ cells are the only PSA producers, we cannot apply adaptive therapy because the PSA never drops to half of its initial value before TCR. The number of treatment cycles in the adaptive therapy protocol, as well as their length, vary depending on *α* and *β*. It is important to note that treatment is continued after reaching TCR. After TCR, we observe oscillations in the population sizes of *T*^*P*^ only if *T*^*P*^ supports PSA production, i.e., if *β* > 0. **F**) Heat map of TCR for different values of *α* and *β*.

Fig 1 illustrates the population size of the three different cell types *T*^+^, *T*^*P*^, and *T*^−^, as well as the total cell count when applying MTD (Fig 1A and 1E) or AT (Fig 1B–1D). Herein, we focus on the best responder scenario. The TCR is highlighted with a black dot and the yellow-shaded area covers the time after competitive release, when the treatment protocol is continued but strategically the treatment has already failed.

We observe that in all cases with *α*, *β* ≠ 0 AT can increase TCR compared to applying MTD. However, if *α* = *β* = 0, we cannot apply AT, as the *T*^−^ cells are not targeted by the treatment and, thus, the PSA level never drops to half of its initial value. Insights into the PSA dynamics in the different cases can be found in Supporting information. Table 2 demonstrates the superiority of AT over MTD: Under AT the TCR is increased by 32%, 14%, and 13%, if only *T*^+^ cells are contributing to the PSA production, only *T*^*P*^ cells are contributing to PSA production, and all three types are contributing to the PSA production equally, respectively.

**Table 2:** Time to competitive release (TCR) for the best responders.

Parameter values	TCR under MTD	TCR under AT	Absolute Improvement	% Improvement
*α* = 1; *β* = 0	4075	5374	1299	32%
*α* = 0; *β* = 1	4075	4665	590	14%
$\alpha =\frac{1}{3}$ ; $\beta =\frac{1}{3}$	4075	4596	521	13%
*α* = 0; *β* = 0	4075	N/A	N/A	N/A

TCR under maximum tolerable dose (MTD) and under adaptive therapy (AT) including the TCR percentage improvement depending on different assumptions on PSA production. Applying AT increases TCR in all cases. We observe the highest improvement in the TCR for *α* = 1, *β* = 0.

The number of treatment cycles as well as their length vary depending on *α* and *β*. In particular, when the PSA production is supported mainly by the *T*^*P*^ cells, we observe shorter treatment cycles leading to higher frequency in the oscillations of the cancer population size. This is caused by the fact that *T*^*P*^ cells are directly targeted by the treatment, resulting in an immediate response in the PSA level if their contribution to the PSA production is high. *T*^+^ cells are only influenced by the treatment via the *T*^*P*^ cells and thus, there is a small delay in the drop of the PSA level, which in turn leads to longer treatment cycles.

If *α* = 0 and *β* = 1, we observe strong oscillations in the population size of *T*^*P*^ cells. As long as enough *T*^*P*^ cells are present and their contribution to the PSA production is high enough, the PSA level can be influenced by the treatment and thus, the AT protocol will lead to continuing treatment cycles after TCR.

While in Fig 1A–1E we focus on the population size dynamics for a few selected values of *α* and *β*, Fig 1F shows a heat map of time to competitive release for all possible values of *α* and *β*.

### Responders

**Fig 2:**
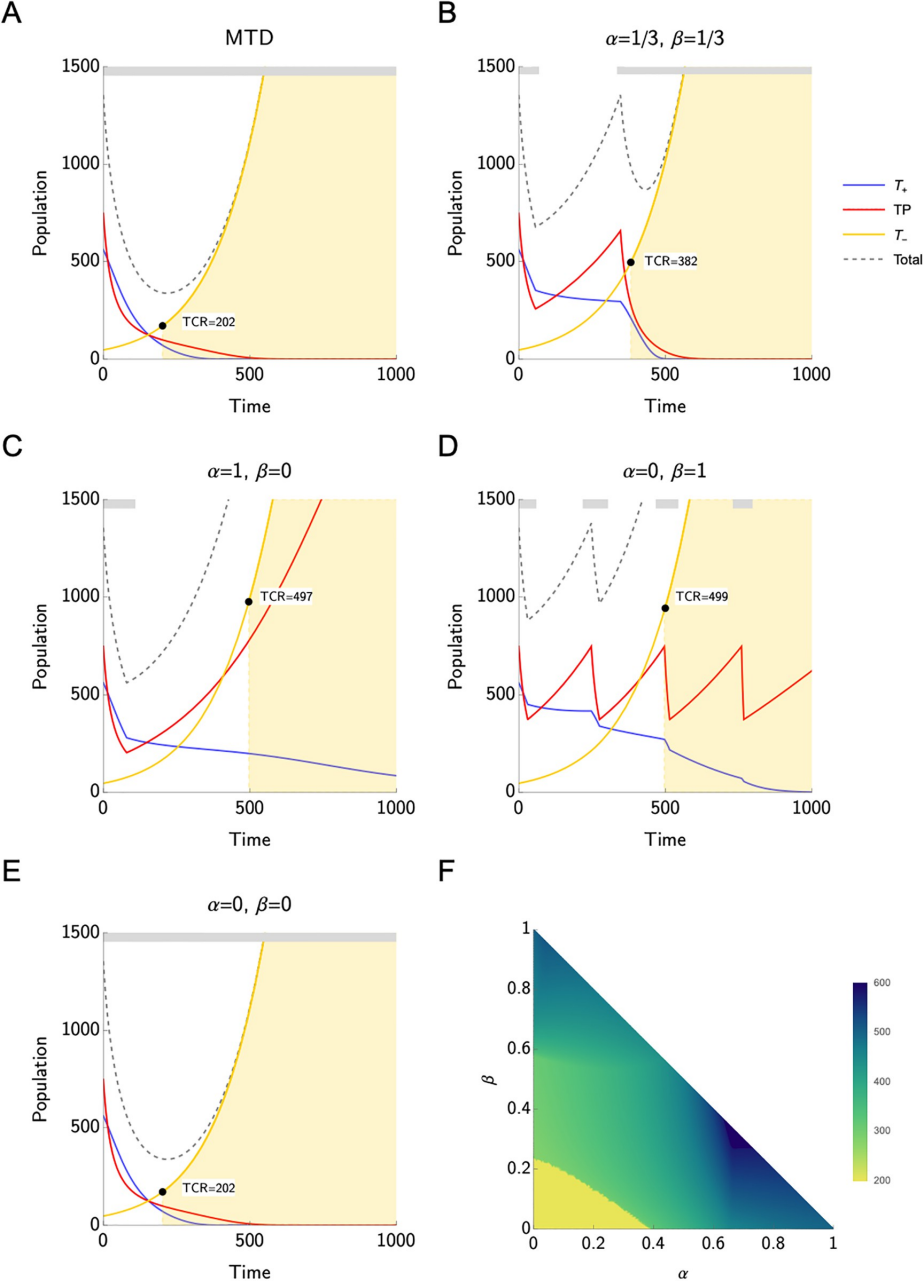
Responders. **A-E**) Time to competitive release (TCR) under maximum tolerable dose (A and E) and adaptive therapy for different values of *α* and *β* (B-D). The grey bar on the top of each plot indicates when treatment is on. For all the values of *α* and *β* considered here, adaptive therapy increases the TCR compared to the standard of care with maximum tolerable dose. The only case where we could not apply adaptive therapy is when *α* = *β* = 0, i.e., *T*^−^ cells are the only PSA producers. This is due to the fact that the treatment does not affect the PSA level in such a case. We observe the highest TCR for *α* = 0, *β* = 1, followed by the case with *α* = 1, *β* = 0, which has a similar TCR. **F**) Heat map of time to competitive release for different values of *α* and *β*.

Fig 2A–2E show the population dynamics for the three cell types in the responder scenario. Also in this scenario, AT increases the TCR with respect to MTD for all considered assumptions on the PSA producers. Thus, the results are qualitatively similar to those of the best responders. However, quantitatively, in this scenario the TCR is much lower than in the previous case, both for MTD and AT. While applying MTD leads to a TCR of 202, the highest TCR corresponds to the cases *α* = 1, *β* = 0 and *α* = 0, *β* = 1 (499 and 497, respectively). As before, we cannot apply AT when *α* = *β* = 0. The results in terms of TCR obtained under different assumptions on *α* and *β* and TCR improvement of applying AT compared to applying MTD are displayed in Table 3.

**Table 3:** Time to competitive release (TCR) for the responders.

Parameter values	TCR under MTD	TCR under AT	Absolute Improvement	% Improvement
*α* = 1; *β* = 0	202	497	295	146%
*α* = 0; *β* = 1	202	499	297	147%
$\alpha =\frac{1}{3}$ ; $\beta =\frac{1}{3}$	202	382	180	89%
*α* = 0; *β* = 0	202	N/A	N/A	N/A

TCR under maximum tolerable dose (MTD) and under adaptive therapy (AT), including the TCR percentage improvement, depending on different assumptions on PSA production. Applying AT increases TCR in all considered cases. We observe the highest improvement in the TCR for *α* = 1, *β* = 0 and a similar improvement for *α* = 0, *β* = 1.

For *α* = 1, *β* = 0, and $\alpha =\beta =\frac{1}{3}$, the AT treatment is stopped before reaching TCR. For *α* = 0, *β* = 1, there are at least two full treatment cycles. However, as expected, the number of cycles here is much lower than in the best responder scenario.

Fig 2F illustrates TCR for all possible values for *α* and *β*. We observe the highest values for TCR if *α* > 0.6, *β* > 0.2 (more details about this case can be found in Supporting information). Interestingly, if the *T*^+^ cells are the only PSA producers, i.e., *α* = 1, the TCR is lower. If the *T*^−^ cells contribute to the PSA production a lot, i.e., *α* < 0.2, *β* < 0.2, the TCR is the lowest (yellow region). That is because in this cases AT can not be applied, as the PSA value never reaches half of its initial size and therefore therapy is never discontinued.

### Non-responders

**Fig 3:**
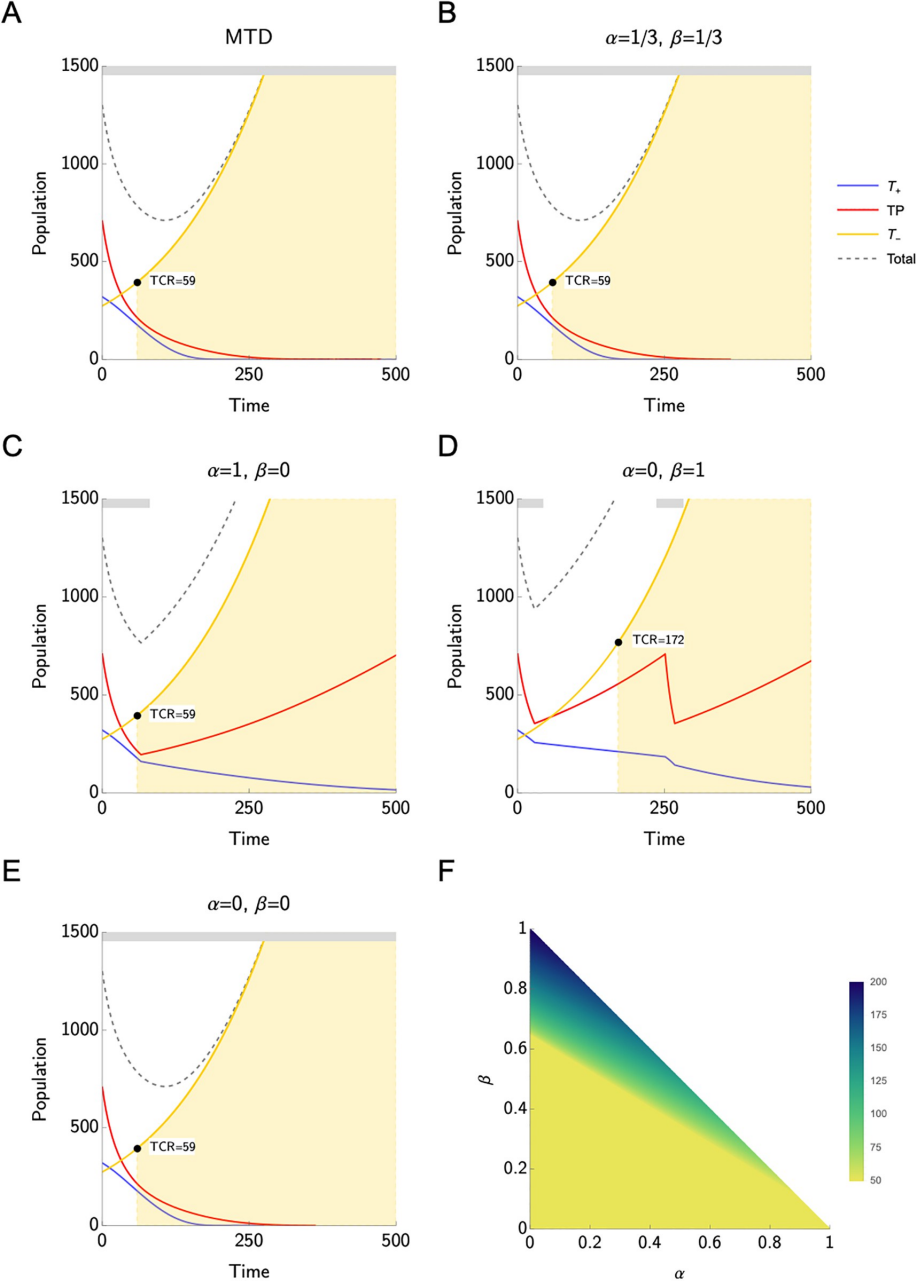
Non-responders. **A-E**) Time to competitive release (TCR) under different assumptions on PSA production. The grey bar on the top of each plot indicates when treatment is on. Adaptive therapy can only be applied if *α* = 0, *β* = 1. In this case, we observe an increase of TCR from 59 (maximum tolerable dose) to 171 (adaptive therapy). In all other cases, adaptive therapy is not applied as the treatment cannot be discontinued before TCR. **F**) TCR heatmap for different values of *α* and *β*.

Fig 3A–3E display the population size dynamics for *T*^*P*^, *T*^+^, and *T*^−^ cells in the non-responder scenario. As expected, the TCR is lower than the TCR of the best responder and responder scenarios. This holds under both AT and MTD. Whenever AT can be applied, i.e., whenever the treatment can be discontinued according to the treatment protocol before TCR is reached, the TCR is increased compared to the standard of care. However, for the combinations of *α* and *β* considered here, it is possible to complete at least one cycle of AT only if *α* = 0, *β* = 1. In this case, i.e., if *T*^*P*^ cells are the only PSA producers, AT can achieve a TCR that is about three times larger than the TCR achieved with the standard of care (see Table 4). Fig 3F supports the results displayed in Fig 3A–3E: The highest TCR can be achieved for *α* = 0, *β* = 1, while in the other three scenarios, the AT cannot be applied.

**Table 4:** Time to competitive release (TCR) for the non-responders.

Parameter values	TCR under MTD	TCR under AT	Absolute Improvement	% Improvement
*α* = 1; *β* = 0	59	N/A	N/A	N/A
*α* = 0; *β* = 1	59	171	112	188%
$\alpha =\frac{1}{3}$ ; $\beta =\frac{1}{3}$	59	N/A	N/A	N/A
*α* = 0; *β* = 0	59	N/A	N/A	N/A

TCR under maximum tolerable dose (MTD) or under adaptive therapy (AT), including the TCR percentage improvement, depending on different assumptions on PSA production. Only if *T*^*P*^ cells are the only cells producing PSA, AT can be applied. In this case, we observe an improvement of 188% in the TCR when applying AT instead of MTD.

## Discussion

PSA is a traditionally used biomarker to track the treatment-induced response in prostate cancer. It has also been used to modulate the treatment in adaptive therapy protocols, such as the one by Zhang et al. [12, 13]. However, its relationship with tumor volume is not well understood. While experimental studies revealed that the production of PSA depends on the tumor composition [30], mathematical models of adaptive therapy usually consider PSA as a surrogate for tumor burden and look only at the total cell count in order to determine when to pause or resume the treatment [12, 16, 18, 33].

Herein we have explored how the assumption that different cancer cell types contribute to the PSA level differently impacts the superiority of adaptive therapy protocols in mCRPC over the standard of care with continuous maximum tolerable dose. We focused on one particular adaptive therapy protocol, i.e. abiraterone therapy on mCRPC [13]. This protocol discontinues abiraterone treatment when the PSA level gets below half of its initial value and abiraterone is re-administered only once PSA recovers to its initial value [12]. We compared time to competitive release (TCR) of Zhang et al.’s adaptive protocol to that of abiraterone maximum tolerable dose when mCRPC is modelled as in [12, 18, 19]. We did this under the assumption that different cancer cell types may contribute to the PSA level differently. For example, in the limit case, we assumed that either *T*^+^ cells, *T*^*P*^ cells, or *T*^−^ cells may be the only PSA producers, while we also analyzed other scenarios, such as those when the three cell types contribute to PSA equally.

We measured time to competitive release for three categories of patients analyzed in [18]: best responders, responders and non-responders, which have no, low, and remarkable initial proportion of abiraterone-resistant *T*^−^ cells, respectively. Moreover, in the model considered here, these three categories are described by different competition matrices, corresponding to very good, intermediate and very bad prognoses, respectively.

Our results show that the Zhang et al.’s adaptive therapy protocol outperforms the standard of care based on maximum tolerable dose whenever adaptive therapy can be applied. The best responders have the longest time to competitive release under MTD compared to the other categories. PSA-guided adaptive therapy can further improve the time to competitive release by 13–32%, depending on the contribution of the different types to PSA production (Fig 1A–1E, Table 2). The greatest improvement is found in the case where the PSA is secreted almost exclusively by the *T*^+^ cells (Fig 1F). When PSA is produced only by the *T*^−^ cells, Zhang et al.’s adaptive therapy cannot be applied, as demonstrated in Fig 1.

For the responders, adaptive therapy can prolong the time to competitive release by 89–147% compared to MTD (Table 3). Similarly to the previous category, the most favourable outcome corresponds to the case where the PSA is secreted (almost) only by the *T*^+^ cells, as shown in Fig 2. When *T*^−^ cells are the only type of cells contributing to the PSA production, Zhang et al.’s adaptive therapy cannot be applied.

As expected, the non-responders have the shortest time to competitive release under MTD when compared to the other categories (Table 4). Fig 3 shows that for this category adaptive therapy can only be applied when the PSA is produced mostly by the *T*^*P*^ cells. In such a case, adaptive therapy can improve the TCR by 188% compared to that of the standard of care.

Overall, adaptive therapy proved to lead to a higher time to competitive release than the standard of care whenever it could be applied.

Gustavsson et al. (2005) investigated PSA secretion in androgen-dependent and independent cells *in vitro* [30]. They reported that the level of PSA secreted by the androgen-dependent cells was tenfold higher than that by androgen-independent cells. This suggests that it might be unlikely to have the *T*^−^ cells as the main PSA producers, which corresponds to the case where adaptive therapy cannot be applied in the categories considered here.

A question remains whether the coefficients *α* and *β* governing the PSA dynamics (Eq 7) can vary with time and/or tumor characteristics.

We did not consider delayed PSA dynamics, as we could not find information on how precisely the dynamics should be delayed and as we confined to the models of [18, 19] here. Identifying realistic PSA dynamics is a subject for future work.

In this work, time to competitive release measured success of the considered therapies, as opposed to a more standard time to PSA progression. While it may be difficult to estimate this time in reality, our approach is more conservative than time to PSA progression. This is because time to competitive release is typically lower than that of the PSA progression [19]. Identifying time to competitive release accurately would open a window of opportunity for patients, as physicians may have time to consider alternative treatment options to delay PSA progression once the competitive release is identified.

We considered therapy resistance as a qualitative trait, as there are two types of cancer cells (*T*^*P*^ and *T*^+^) which are targeted by the therapy and one type (*T*^−^) which does not respond, neither to androgen deprivation nor abiraterone treatment as it is independent of testosterone. This can be expanded to the situation where multiple drugs are applied and phenotypes resistant to more drugs at the same time are present [34]. Conversely, some recent works considered quantitative resistance [32, 35, 36]. In any case, even with resistance as a quantitative trait, Zhang’s treatment protocol would be more successful than MTD [37].

Since Zhang et al.’s trial, different evolutionary cancer treatment protocols, i.e. protocols that anticipate and steer eco-evolutionary cancer dynamics, have been proposed in the literature [16, 32, 35–39] and their impact on patients’ quality and quantity of life has been analyzed also theoretically [39–42]. For example, Viossat and Noble (2021) demonstrated that already discontinuing the treatment when PSA reduces to a higher proportion (for example 80%) of its initial value would be more effective than the original Zhang et al.’s protocol where treatment is continued until PSA drops by half. Cunningham et al. (2020) demonstrated that the mCRPC tumor burden may be stabilized through a dose titration protocol [19]. Gatenby et al. (2019) suggested that cure in mCRPC may be possible if a different therapy is applied in a strategic way when the initial treatment response is observed [38]. The fact that the classical adaptive therapy protocol that we analyzed here performs very well under the vast majority of assumptions on the contribution of different cell types’ to the prostate specific antigen level is a very good news for both these other evolutionary therapies and patients with metastatic disease, as long as PSA remains the main marker for tumor progression. To further improve therapy design in mCRPC, a better understanding of mechanisms behind the PSA production and/or alternative biomarkers in mCRPC are needed [43, 44]. For instance, combining the monitoring of the PSA with the evaluation and quantification of bone metastasis by bone scintigraphy as well as other imaging studies would contribute to optimizing the treatment strategy [45]. More effective methods to estimate the changes in the tumor burden might be circulating tumor cells, recently used in clinical trials [46]. Combining these options with our predictive mathematical models will help to answer key questions in the ecology and evolution of cancer, such as “How can game theory be utilized to understand tumorigenesis and potentially guide therapy?” or “Are there measures of the evolution and ecology of tumours that can be used to develop a classification system for tumours, so as to improve prediction, prognosis and management of tumours?” [47]. More importantly, such a development will likely improve odds for patients suffering the metastatic disease.

## Supporting information

S1 Fig The PSA dynamics of the best responder patient for different values of *α* and *β*. For the case *α* = 1/3, *β* = 1/3 the PSA represents an average of the three populations, for the case *α* = 1, *β* = 0, the PSA mesures only the *T*^+^, for the case *α* = 0, *β* = 1 it measures the *T*^*P*^, while for the case *α* = 0, *β* = 0 it corresponds to the *T*^−^.
(PNG)

S2 Fig The responder case for *α* = 0.7, *β* = 0.3. This case is very similar to the case *α* = 1, *β* = 0, with only a minor improvement in terms of time to competitive release. This is because the parameters allow for higher proportions of *T*^+^ and *T*^*P*^, which delay a bit the growth of the *T*^−^.
(PNG)

## Abbreviations

The following abbreviations are used in this manuscript:

AT: Adaptive therapy
mCRPC: Metastatic Castrate-Resistant Prostate Cancer
MTD: Maximum tolerable dose
PSA: Prostate-specific antigen
TCR: Time to competitive release
