# Supplementary figures and images for: The effect of tumor composition on the success of adaptive therapy: The case of metastatic Castrate-Resistant Prostate Cancer
Source: PLoS One. 2024 Sep 26;19(9):e0308173. doi: 10.1371/journal.pone.0308173 (PMC11426540; doi:10.1371/journal.pone.0308173)

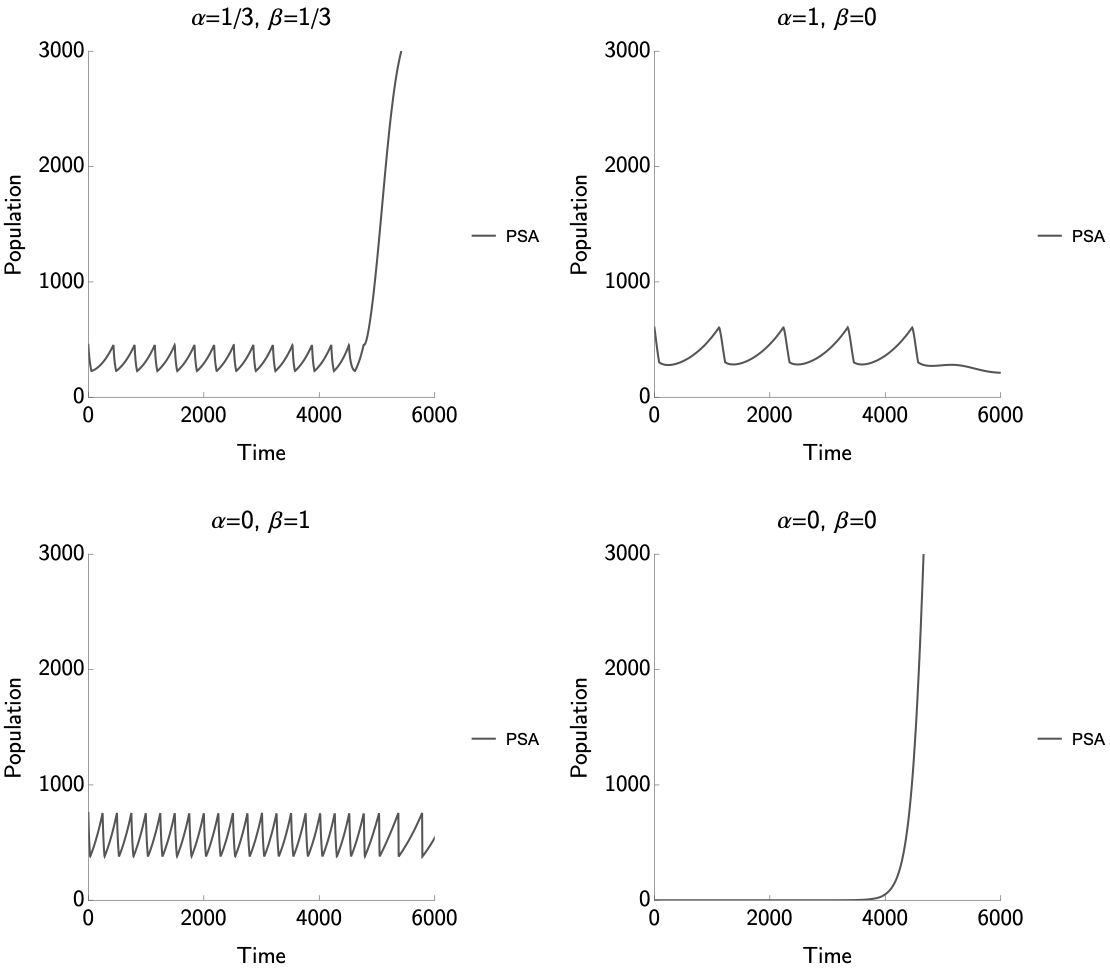

Supplement: S1 Fig — For the case α = 1/3, β = 1/3 the PSA represents an average of the three populations, for the case α = 1, β = 0, the PSA mesures only the T+, for the case α = 0, β = 1 it measures the TP, while for the case α = 0, β = 0 it corresponds to the T−. (PNG) [file pone.0308173.s001.png]

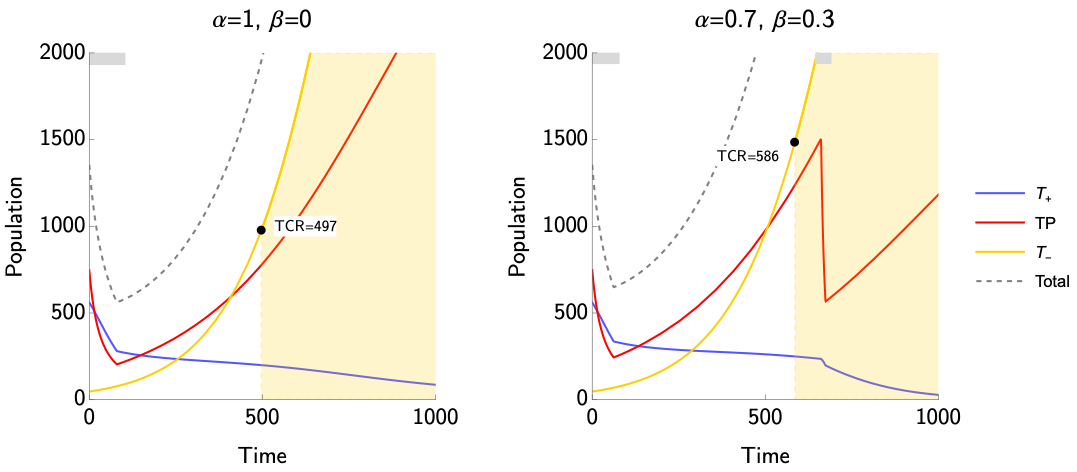

Supplement: S2 Fig — This case is very similar to the case α = 1, β = 0, with only a minor improvement in terms of time to competitive release. This is because the parameters allow for higher proportions of T+ and TP, which delay a bit the growth of the T−. (PNG) [file pone.0308173.s002.png]
